# The Potential of Integrative Cancer Treatment Using Melatonin and the Challenge of Heterogeneity in Population-Based Studies: A Case Report of Colon Cancer and a Literature Review

**DOI:** 10.3390/curroncol31040149

**Published:** 2024-04-03

**Authors:** Eugeniy Smorodin, Valentin Chuzmarov, Toomas Veidebaum

**Affiliations:** 1 Department of Chronic Diseases, National Institute for Health Development, Paldiski mnt 80, 10617 Tallinn, Estonia; toomas.veidebaum@tai.ee; 2 2nd Surgery Department, General Surgery and Oncology Surgery Centre, North Estonia Medical Centre, J. Sütiste Str. 19, 13419 Tallinn, Estonia

**Keywords:** melatonin, cancer, integrative treatment, zinc, selenium, vitamin D, green tea, taxifolin, aspirin, inflammatory markers, hemostasis, N-of-1

## Abstract

Melatonin is a multifunctional hormone regulator that maintains homeostasis through circadian rhythms, and desynchronization of these rhythms can lead to gastrointestinal disorders and increase the risk of cancer. Preliminary clinical studies have shown that exogenous melatonin alleviates the harmful effects of anticancer therapy and improves quality of life, but the results are still inconclusive due to the heterogeneity of the studies. A personalized approach to testing clinical parameters and response to integrative treatment with nontoxic and bioavailable melatonin in patient-centered N-of-1 studies deserves greater attention. This clinical case of colon cancer analyzes and discusses the tumor pathology, the adverse effects of chemotherapy, and the dynamics of markers of inflammation (NLR, LMR, and PLR ratios), tumors (CEA, CA 19-9, and PSA), and hemostasis (D-dimer and activated partial thromboplastin time). The patient took melatonin during and after chemotherapy, nutrients (zinc, selenium, vitamin D, green tea, and taxifolin), and aspirin after chemotherapy. The patient’s PSA levels decreased during CT combined with melatonin (19 mg/day), and melatonin normalized inflammatory markers and alleviated symptoms of polyneuropathy but did not help with thrombocytopenia. The results are analyzed and discussed in the context of the literature on oncostatic and systemic effects, alleviating therapy-mediated adverse effects, association with survival, and N-of-1 studies.

## 1. Introduction

The hallmarks of cancer are genetic deregulation and epigenetic modifications caused by external and internal factors, which lead to abnormal cell growth and dissemination. A growing tumor creates a surrounding vasculature necessary for nutrition under conditions of hypoxia and deregulated metabolism and interacts with other cells in the microenvironment [1]. High heterogeneity, plasticity, and self-renewal help tumor cells avoid immune surveillance, become resistant to chemotherapy (CT) and apoptosis, and spread, creating metastatic niches. A developing tumor has both local and systemic effects on the body, which leads to cachexia, psychoneuroendocrine and immunoinflammatory imbalances, pro-tumoral cytokine production, aberrant myelopoiesis, immunosuppression, chronic inflammation, paraneoplastic syndromes, hemostatic abnormalities, and other disorders [2,3,4,5,6].

Due to the unsatisfactory efficacy and adverse effects of conventional therapies in advanced cancer, some patients themselves use additional or alternative treatments that can be beneficial, not helpful, or harmful. A patient’s response to treatment depends on the characteristics of the tumor and the patient’s state of health. Poor sleep, an unbalanced diet, distress, and lack of physical activity can significantly affect outcomes and life expectancy. All these factors can be improved by a patient through self-management, following the recommendations of oncologists and healthcare experts. In this regard, an integrated approach to treatment and improvement in overall health and quality of life is becoming increasingly important for personalized oncology [7]. Despite advances in conventional treatment, current therapies are insufficient to control metastatic disease. Colorectal cancer (CRC) continues to be the third-most diagnosed type of cancer but second in terms of mortality in 2020 [8]. Acquired resistance of tumors to CT, the harmful effects of CT and radiation therapy (RT), and the high cost and limitations of targeted therapy and immunotherapy (IT) stimulate the search for new treatment strategies [9,10,11,12]. To improve patient outcomes, a therapeutic approach using a combination of natural products that can simultaneously act on various pathways of tumorigenesis is attracting attention [13].

Melatonin (Mel) is involved in many vital cellular functions and affects the epigenetic mechanisms, gene expression, and signaling pathways associated with the emergence and progression of cancer. Mel is an immunomodulator and one of the transcriptional modulators affecting tumor homeostasis [14,15]. Mel, nutrients (zinc, selenium, and vitamin D), and plant polyphenols may be useful supplements in the treatment of cancer and require additional clinical trials [16,17,18,19,20], but the methods and doses used in many preclinical studies are inadequate for translation to clinical settings. Parallel randomized and placebo-controlled trials (RCTs) have encountered obstacles to resolving the contradictory results of adjunctive treatments due to the heterogeneity associated with many factors, including poor patient stratification, individual response to treatment, bioavailability, and the complex multifaceted relationship between a tumor and homeostasis. A personalized approach based on testing clinical parameters in response to integrative treatment in dynamic, patient-centered N-of-1 studies deserves greater attention. Integrative treatment with Mel directed to systemic effects in cancer and adverse consequences of conventional therapy may improve quality of life and prolong overall survival, as shown in some preliminary clinical studies discussed below. The present paper describes the dynamics of the clinical parameters of a patient with colon cancer who took Mel, nutrients, and aspirin. Integrative treatments are analyzed and discussed in the context of a review of the literature examining their oncostatic and systemic effects as well as the influence on conventional therapy and survival. Monitoring of clinical parameters before, during, and after CT with Mel as well as their comparison when taking Mel and its withdrawal may be considered an example for N-of-1 study designs of personalized responses to integrative treatment with Mel.

## 2. Materials and Methods

This analysis and monitoring of clinical parameters were based on patient protocols and laboratory tests at the North Estonia Medical Centre. An integrative treatment with Mel and nutrients was applied by the patient on his own initiative, and the design was based on the scientific literature and his qualifications and competence (Ph.D., biochemistry, pharmacology, and oncology). Doses of Mel and the period of its administration and withdrawal were selected by the patient himself. Investigations were carried out following the rules of the Declaration of Helsinki and in accordance with the ICH GCP Standards. The literature review was compiled using information from PubMed, Web of Science, Google Scholar, and Crossref. Based on the results of the completed study, this paper was written taking into account the patient’s consent.

## 3. Results

### 3.1. Colonoscopy, Computed Tomography, and Histology

A 69-year-old nonsmoking man was diagnosed with a malignant tumor of the sigmoid colon and two polyps during colonoscopy. His parents had no history of gastrointestinal cancer but were diagnosed with ovarian and prostate cancer. In July 2018, he was operated on. The distal part of the colon with a tumor along with the surrounding lymph nodes was removed. Tubular adenocarcinoma with tubular adenoma and hyperplastic polyps were found in biopsies and resected tumors. In addition, subserous tumor deposits in adipose tissue were found in the specimens. The tumor was classified as pT3N1c (Nx) M0 G2, stage III B. Immunohistochemical analysis revealed 80% for Ki-67 and 90% for p53. Metastases in the lung, liver, and abdominal cavity were not detected in preoperative computed tomography and twice after surgery for 4 years. During a third colonoscopy, one hyperplastic polyp was removed.

In cancer pathology, tumor deposits are defined as focal aggregates of cancer nodules located in the pericolonic or perirectal adipose tissue or adjacent mesentery separately from the invasive margin and lymph node tissue. The presence of tumor deposits is an independent unfavorable prognostic factor for patients with sigmoid colon cancer. Adjuvant CT can significantly improve survival in patients with subcategory N1c colon cancer [21,22].

Ki-67 is a nuclear protein associated with cell proliferation. A high percentage of Ki-67-expressing cells in the tumor material is an unfavorable prognostic factor for CRC patients [23]. However, the prognostic role of Ki67 in CRC remains controversial, which can be explained by a better response to adjuvant therapy in patients with a high tumor cell proliferation index. Rapidly proliferating cancer cells may be more vulnerable to adjuvant therapy. A favorable prognosis has been reported in patients having tumors with higher Ki-67 expression who underwent both surgery and adjuvant radiochemotherapy but not in patients who underwent surgery alone [24,25].

The p53 regulatory protein is a transcription factor that acts as a tumor suppressor. It can induce cell cycle arrest to repair DNA damage, induce apoptosis, block proliferation, and inhibit tumor angiogenesis. In addition, p53 is activated in response to stress factors, and it regulates various pathways inherent in oncogenesis. The *TP53* gene is the most frequently mutated gene (>50% in human cancer cells). In general, patients with abnormal p53 have an increased risk of death both when assessing p53 protein accumulation and when analyzing mutations [26]. Mutations in *TP53* are more likely to be associated with the risk of metastasis than immunohistochemically assessed accumulation of p53 protein in the primary tumor. However, mutations in *TP53* can have different effects, including gain or loss of function, and affect the sensitivity of tumors to treatment in different ways [26]. CT and RT can also induce different mutations in tumor cells. Abnormal accumulation of p53 protein in tumors may also have a complex relationship with the tumor response to cytotoxic drugs and radiation. This may be one of the reasons for the contradictory results in studies regarding the benefit of CT in patients with overexpression of the p53 protein [26,27,28,29].

### 3.2. Systemic Chemotherapy

Eight cycles of CT were prescribed, but due to thrombocytopenia, four cycles of CAPOX with oxaliplatin (100–130 mg/m^2^) and capecitabine (2–3 g per day) along with antiemetics were performed. Adverse effects were observed, such as laryngopharyngeal dysfunction, numbness and tingling in the hands that occur with cold exposure, diarrhea, nausea, anemia, thrombocytopenia, and neutropenia. The adverse effects of CT were temporary and gradually disappeared. CT was suspended after the first cycle due to sinusitis and bronchitis, which were treated with antibiotics. Oral amoxicillin (2 g/day, 7 days) was ineffective; therefore, azithromycin (250 mg/day, 5 days) was administered orally, which was more effective.

CT with oxaliplatin/capecitabine causes neutropenia, lymphocytopenia, and thrombocytopenia [30]. Oxaliplatin is one of the most neurotoxic anticancer drugs, causing long-term peripheral neuropathy [31]. After completion of CT, the patient did not have any residual manifestations of neuropathy. Two months after CT, numbness and decreased sensation were noted in the toes and soles. Melatonin can promote neuritogenesis in oxaliplatin-challenged cells but does not interfere with the cytotoxic activity of oxaliplatin in a human colon cancer cell line; it can also reduce neuropathic manifestations [32,33,34]. The use of Mel or its combination with zinc [35] during and after chemotherapy could reduced and delayed the onset of CT-mediated neurological adverse effects in the patient.

### 3.3. Clinical Manifestations in Using Melatonin

The patient used a sublingual form of Mel (Melatosell Strong, Hankintatukku OY, Karkkila, Finland) in the evening and at night during the first awakening for 11 months, including doses of 1.9–5.7 mg/day a month before surgery and 5.7–7.6 mg/day after surgery. He took a dose of 19 mg/day during CT and 9.5 mg/day between cycles of CT. After a 1-year break, the patient resumed taking Mel at lower doses (1.9–3.8 mg/day) at 1-month intervals, and then at 1–2-week intervals. According to his subjective assessment, mild to moderate effects, such as sedation, intermittent sleep, and daytime drowsiness were noted. The use of Mel after diagnosis promoted relaxation, alleviated stress, anxiety, and depressive symptoms, thereby promoting falling asleep and longer sleep. Mel withdrawal did not cause insomnia or addiction.

### 3.4. Aspirin, Food Supplements, and Nutrition

The patient started taking low-dose aspirin (Hjertemagnyl, Takeda, Oranienburg, Germany, 75 mg daily) 8 months after surgery, according to its benefits and harms in an updated modeling study and results of a systematic review and meta-analysis [36,37]. The patient, for a long time before and after diagnosis, took selenium (Se) and zinc supplements at a daily dose of 50 μg of Se (selenomethionine and sodium selenite) and 10–15 mg of zinc. In addition, he took manganese, magnesium, and vitamins D3, B6, and C in generally accepted doses. Magnesium and B6 were used to reduce muscle cramps at night. The daily dose of Se was temporarily increased to 200 μg/day in the last cycle of CT and for 6 weeks after CT. The serum level of Se also showed an increase from 121 to 150 µg/L (analysis in Synlab). Optimal plasma Se values have been estimated at 90–120 µg/L [38], but higher Se levels might be associated with a reduced risk of certain cancers and better survival in those with CRC [39,40]. The patient was taking vitamin D3 (600–2500 IU) to maintain normal plasma levels of its metabolite calcitriol, which ranged from 63 to 104 nM. In addition, he took probiotics and prebiotics to normalize bowel function as well as natural polyphenols (after chemotherapy, periodically, with an interval of 1 month), namely, taxifolin (dihydroquercetin, 25 mg) and 600 mg of green tea extract (Evalar). The tablets were stored in a minimum volume of olive oil at 4 °C to protect against oxidation and improve the bioavailability of polyphenols [41].

The patient adhered to an active lifestyle and rational nutrition, according to beneficial recommendations [42,43,44,45,46,47]. He excluded roast, red, and processed meats, reduced the consumption of fatty and sweet foods, granting preference to dairy products, yogurts, fish, nuts, cereals, vegetables, fruits, olive and flaxseed oils, fish oil, and dietary fibers. The patient did not use other seed oils with an increased content of proinflammatory and pro-tumorigenic linoleic acid (omega-6 polyunsaturated fatty acid) [48,49,50,51]. He consumed, in increased amounts, berries containing polyphenols (bilberry, cranberry, etc.), green tea, and ginger, while alcohol consumption was kept to a minimum. A low body mass index may be a significant negative predictor of outcome in patients with CRC [52]. The patient lost 10% of his weight and, despite a normal appetite, did not regain his original weight. Taking melatonin may influence body weight [53].

Integrative treatment using dietary supplements and antioxidants can reduce the toxic and harmful effects of CT and RT on the body. On the other hand, together with conventional therapy, they can affect the effectiveness of therapy by removing therapy-induced cytotoxic reactive oxygen species (ROS) and influencing drug metabolism [54,55,56]. Opinions on the benefits or risks of antioxidants vary, and it is difficult to assess the overall outcome [54,57]. The category of “antioxidants” studied in reviews and meta-analyses includes substances with different mechanisms of action on normal and tumor cells, which are not limited to ROS removal. For example, polyphenols are being investigated as epidrugs that affect the epigenome program, thereby restoring cellular memory in cancer [1]. The antioxidant and prooxidant effects of Mel on human cell lines have been shown, depending on the concentration and duration of exposure. Mel can promote the formation of ROS and the death of some cancer cells as well as enhance the cytotoxic effect of drugs on tumor cells [58].

### 3.5. Clinical Parameters and Their Dynamics

#### 3.5.1. Tumor-Associated Systemic Inflammation

Systemic and local inflammation are considered as key elements in the development of cancer since inflammation drives tumor initiation, growth, progression, and spread [4,59,60]. Numerous studies collected in reviews [61,62] demonstrate that routinely used biomarkers associated with inflammation, namely, the ratio of neutrophils/lymphocytes (NLR), lymphocytes/monocytes (LMR), and platelets/lymphocytes (PLR), can be applied for prognostic CRC assessment. The risk of mortality has been found to increase in patients with a higher NLR and PLR and a lower LMR. This association can be considered as unfavorable systemic inflammation and, in terms of immunosuppression, causes a decrease in the lymphocyte population [4,60,63,64,65].

Reported values of prognostic sensitivity and specificity can be assessed as moderate or high, but the cut-off values and AUC (area under the curve) vary [61,62]. In addition, values of the ratios require unification since they are influenced by tumor localization, pathological data, and concomitant non-cancer diseases.

Postoperative inflammation-based prognostic markers can more accurately predict overall survival and recurrence-free survival in patients with stage III CRC [66]. The dynamics, association with survival, and clinical relevance of the ratios were studied by Herold et al. [67]. The authors note that personalized temporal changes in clinical parameters may more accurately reflect the risks of cancer progression. Furthermore, based on a prognosis-related blood cell count, an integrated prognostic inflammatory index with optimal cut-offs and nomograms was developed for personalized prediction of CRC survival [68].

**Figure 1:**
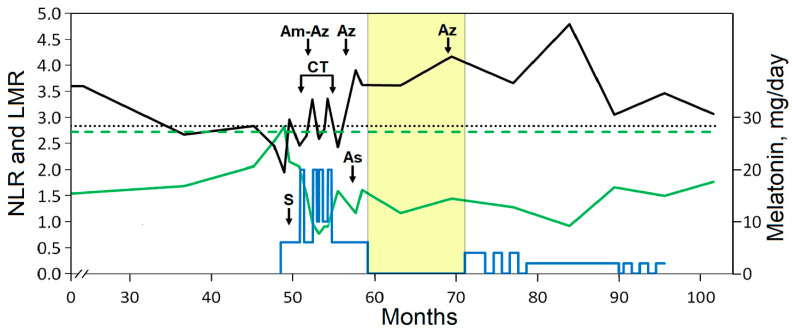
Dynamic study of the ratio of neutrophils/lymphocytes (NLR, green line) and lymphocytes/monocytes (LMR, black line). The blue line indicates the doses of Mel supplementation. Arrows indicate the time of surgery (S) and the chemotherapy (CT) interval as well as intake of aspirin (As) and prescribed antibiotics: amoxicillin and then azithromycin (Am-Az) or azithromycin alone (Az). Cut-off values for NLR (adverse at >2.72, dashed green line) and LMR (adverse at ≤2.83, dotted line) were taken from reference [69]. The yellow area indicates the withdrawal interval of Mel intake.

**Figure 2:**
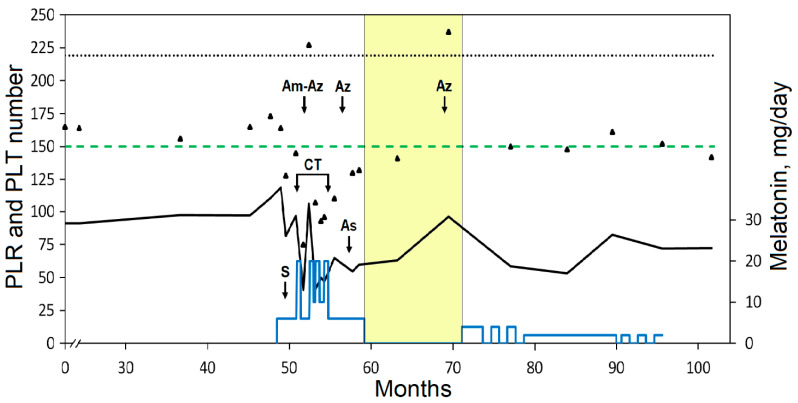
Dynamic study of the ratio of platelets/lymphocytes (PLR, black line) and platelets (PLT, triangles). Designations are the same as in Figure 1. The cut-off value for PLR (adverse at >219, dotted line) is taken from reference [69]. The dashed green line indicates the lower limit of the reference range for PLT. The blue line indicates the doses of Mel supplementation. The yellow area indicates the withdrawal interval of Mel intake.

The patient’s follow-up study showed that the preoperative NLR was slightly above the cut-off but decreased significantly after surgery and remained below the cut-off. The low preoperative LMR varied after surgery and during CT and then increased and remained above the cut-off (Figure 1). The low value of PLR associated with a low platelet (PLT) count remained below the cut-off (Figure 2). The cut-offs presented in Figure 1 and Figure 2 were selected based on the study with largest number of participants (5336) and with the highest proportion of patients with stage III CRC (48.3%) compared to other similar studies [69]. CT caused transient neutropenia, while the count of lymphocytes remained within the reference range. Notably, the reduced count of lymphocytes gradually increased after surgery and even during and after CT (Table 1). This was reflected in the lower NLR and higher LMR and may be related to the beneficial effect of Mel. Preliminary clinical studies have shown that the use of Mel can increase the LMR value that can potentially be used in antitumor IT [70,71]. A low lymphocyte count during chemotherapy is an unfavorable factor as it may predict worse disease-free survival in patients with colon cancer [72]. Tumor infiltration of T lymphocytes is an independent informative prognostic factor that was confirmed in a systematic review and meta-analysis of patients with CRC [73].

Mild anemia and preoperative eosinopenia were observed in the patient. Anemia was not associated with Mel use. The number of eosinophils significantly increased after surgery and remained within the reference range (Table 1). A decrease in the number of circulating eosinophils in patients with CRC has been associated with a shorter overall and disease-free survival [74]. Based on the prognostic value of eosinophil- and basophil-related markers, a scoring system and nomogram for predicting overall survival and individualized risk stratification of patients with CRC has been created [75].

#### 3.5.2. Tumor Markers

**Figure 3:**
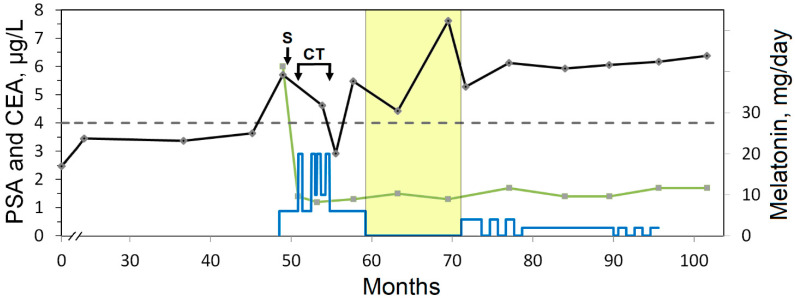
Dynamic study of tumor markers: PSA (black line) and CEA (green line). The designations are the same as in Figure 1. The dashed line indicates the upper limit of the reference range for PSA. The blue line indicates the doses of Mel supplementation. The yellow area indicates the withdrawal interval of Mel intake.

The preoperative levels of CEA and CA 19-9 decreased after tumor removal and remained within the reference ranges during the follow-up (Table 1, Figure 3). Prostate adenoma had been diagnosed earlier in the patient. An elevated PSA level was observed before surgery; however, the PSA level decreased temporally during CT in combination with Mel. By the end of the withdrawal of Mel after CT, PSA reached a maximum level of 7.61 μg/L, which decreased to 5.28 after Mel was resumed (3.8 mg per day) (Table 1, Figure 3).

#### 3.5.3. Hemostasis Disorders

Venous thromboembolism (VTE) is one of the most common causes of death and morbidity in cancer patients, and CT and immobilization contribute to an increased risk of VTE [76]. The prevalence of coagulopathy, including thrombocytopenia and APTT prolongation, has been shown in patients with CRC [6]. The elevated preoperative and postoperative level of D-dimer (the product of degradation of fibrin by fibrinolysis-mediated plasmin cleavage) and APTT may be risk factors associated with thrombotic events and bleeding, worsening the prognosis of CRC [77,78,79,80]. Cumulative incidences of VTE occur in 3.6% of CRC patients [81]. According to the Khorana scale, CRC is ranked as a cancer with a low thrombogenic risk among other types of organ-specific cancer; however, other authors believe that CRC has a higher thrombogenic risk [81,82]. The D-dimer test reflects the activation of fibrinolysis and thrombosis and is commonly used to evaluate patients with suspected deep venous thrombosis, pulmonary embolism, or disseminated intravascular coagulation. Testing of D-dimer in cancer patients has limitations since surgery, trauma, hemorrhage, infection, and other clinical conditions also increase its level [83]. Elevated levels of D-dimer are frequently observed in cancer patients; however, the test has lower specificity and a higher false-positive rate for VTE in patients compared to the general population. In addition, the clinical utility of D-dimer testing, as measured by the proportion of patients in whom VTE can be ruled out as a D-dimer-negative result, is much lower in cancer than in the non-cancer population. D-dimer levels increase with age, reducing the specificity of testing and requiring the use of an age-adjusted D-dimer cut-off. Thus, most cancer patients need additional diagnostic imaging to rule out VTE [83].

**Figure 4:**
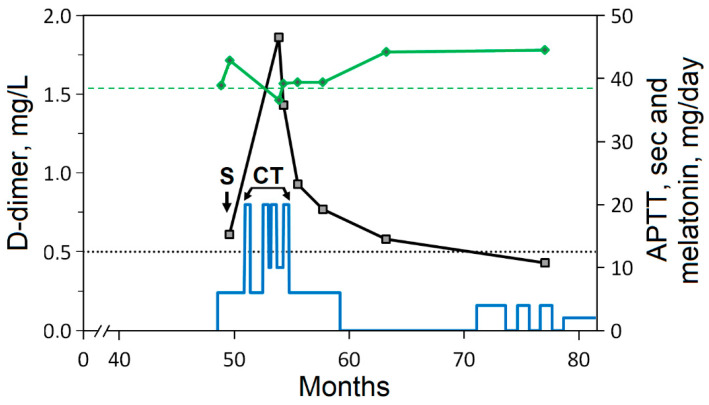
Dynamic study of the level of D-dimer (black line) and the activated partial thromboplastin time (APTT, green line). The designations are the same as in Figure 1. The upper limits of the reference range for D-dimer (dotted line) and APTT (dashed green line) are shown. The blue line indicates the doses of Mel supplementation.

In the present study, the patient did not receive anticoagulant therapy, except for injections of low-molecular-weight heparin within 5 days after surgery for thromboprophylaxis [84]. The elevated postoperative level of D-dimer in the plasma increased during CT with Mel and then gradually decreased to the reference range (Figure 4). The APTT values tended to be above the upper limit in the follow-up. The plasma levels of fibrinogen and antithrombin III and the prothrombin time remained within the reference range. Ultrasonography of the patient did not find thrombotic events.

The influence of Mel on hemostasis and PLT is still poorly understood, and published data are contradictory. Administration of high doses of Mel can reduce thrombosis and levels of plasma fibrinogen but not D-dimer levels, as has been shown in patients infected with COVID-19 and in patients with hemorrhagic stroke [85,86]. In an experimental model, Mel was found to be able to shorten the APTT, prothrombin time, and thrombin time by modulating blood coagulation pathways [87]. Mel can increase the PLT count, including in patients receiving CT [88,89,90]. On the other hand, possible consequences of uncontrolled use of Mel have been reported since Mel can promote PLT aggregation and apoptosis [91]. A decrease in the number of PLTs was observed during CT in combination with Mel; therefore, CT was suspended (Figure 2). The patient was prescribed amoxicillin and azithromycin for the treatment of sinusitis and bronchitis. After treatment with antibiotics, a sharp but transient increase in the number of PLTs was observed (Figure 2, arrows Am-Az and Az). An increase in PLTs after taking antibiotics was noted three times: in the period between interrupted CT cycles and then after chemotherapy, including the period of Mel withdrawal. This is one of the side effects of antibiotics [92,93], which in the present case facilitated the prescription of subsequent CT cycles. The use of antibiotics to treat infections may increase the risk of venous thrombosis [94].

Antibiotics may be prescribed since infection is one of the concomitant diseases in cancer patients. Considering the essential role of the gut microbiota in the development and progression of colon cancer, antibiotics should be used with caution, as they can cause cancer-promoting dysbiosis and affect the efficacy and toxicity of CT by disturbing the balance and diversity of gut bacteria [95,96]. Both CT and antibiotics significantly affect the composition of intestinal bacteria, and bacteria can also influence the response to CT and IT [95,97]. Antibacterial activity against cancer-promoting *Fusobacterium nucleatum* has been found for 5-fluorouracil and aspirin, and individuals who use aspirin daily have a lower bacterial load in colon adenoma tissues [97,98]. This bacterium is an oral pathogen related to periodontal disease and can colonize and predominate in CRC tissue. This is associated with worse overall and cancer-specific survival [99,100]. Oral pathogenic bacteria may contribute to the progression of gastrointestinal cancer through the mechanisms of inflammation, tumor colonization, and dysregulation of the immune response [99]. In the present clinical case, the patient benefited from a publication on the effectiveness of Mel in maintaining oral health [101]. According to his own observations, periodontal inflammation was reduced after regular topical oral use of Mel.

## 4. Discussion

### 4.1. Relevance of the Use of Melatonin in Integrative Cancer Treatment

#### 4.1.1. Physiological Function of Melatonin

The circadian system coordinates vital physiological functions and is synchronously connected with the visual perception of light and dark through the suprachiasmatic nucleus. Physiological functions of the body, including the normal functioning of the gastrointestinal tract, are maintained through circadian rhythms, the desynchronization of which can lead to gastrointestinal disorders and increase the risk of cancer [16,102,103]. Mel functions as a cellular protector and multifunctional hormone regulator that maintains homeostasis through vital circadian rhythms to protect the body from the development of different diseases [104]. Some authors consider Mel as the “cornerstone” on which neuroimmunoendocrinology has been built as an integral concept of homeostasis regulation [105]. Endogenous Mel is produced by the pineal gland mainly at night as well as by other organs, especially the gastrointestinal tract [106]. Mel regulates colonic motility, and the effect of exogenous Mel may be dose-dependent [106,107]. Its potential for the treatment of symptoms of irritable bowel syndrome and ulcerative colitis is being considered, although reports are conflicting. The treatment potential of Mel may be greater for gut disorders exacerbated by circadian disruption [107]. The physiological role of Mel and its therapeutic potential for gut diseases have been discussed in reviews [105,106,108].

Mel can potentially suppress cancer initiation, as a significant negative association between the use of Mel in older adults and the risk of CRC was documented in a population-based cohort study [109]. The disrupted sleep and circadian rhythms in cancer patients is closely associated with poor prognosis and treatment failure [110,111]. Since most cancer patients may suffer from various sleep disruptions, diagnosis and treatment of this comorbidity should not be ignored. Circadian modulation, sleep medication, and sleep-correcting interventions through a healthy daytime lifestyle should become an integrated practice in oncology [110]. Cortisol is a steroid hormone that is also considered a circadian hormone and is produced in response to stress. Cortisol is produced primarily in the adrenal cortex as well as in other tissues but in smaller quantities. Like Mel, cortisol is a multifunctional hormone regulator that maintains homeostasis. Circadian cortisol production may be disrupted in cancer, and the serum Mel/cortisol ratio may be reduced in patients with advanced cancer [110]. Poor sleep may lead to elevated serum cortisol levels and cortisol-mediated immunosuppression, but long-term disruption of circadian rhythms may deplete adrenal cortisol production. The effects of cortisol treatment and how it relates to circadian resynchronization remain unclear and deserve further research [110]. Distress and disruption of sleep and circadian rhythms contribute to tumor progression, as they deplete resources and cause disturbance of immune homeostasis, including conditions associated with dysbiosis and comorbidities [108]. The amplitude of the circadian rhythm in Mel secretion may be decreased in patients with CRC [112]. Regarding the effect on sleep and circadian rhythms, effective doses may be less than 2–3 mg/day, as administration of higher doses may cause the desensitization of Mel receptors [113,114].

#### 4.1.2. Anticancer Activity of Melatonin and Its Preliminary Clinical Studies

The anticancer potential of Mel has been shown in many experimental works [16,17,115,116]. The mechanisms of antitumor activity of Mel are associated with pleiotropic effects on cancer cells and the tumor microenvironment, including the induction of apoptosis; anti-inflammatory, anti-angiogenic, antiproliferative, and antimetastatic action; and the regulation of the immune and psychoneuroendocrine systems [33,71,104,115,117,118,119,120,121,122,123,124,125,126,127,128].

Considering the information obtained in preclinical models, the doses and methods used are generally not adapted for clinical translation. In in vivo experiments, the doses of Mel generally exceed the doses acceptable for humans [129]. Due to poor solubility in aqueous media and the instability of Mel, the true concentrations of Mel tested in vitro can be significantly lower than those presented in some studies (1–10 mM) [129].

Preliminary clinical studies have shown that the use of Mel reduces the harmful effects of CT and RT [115,118,119,120,130,131,132]. In terms of protection against ionizing radiation, the use of Mel can be recommended in regular examinations using computed tomography [133]. According to some preliminary reports, Mel reduces the incidence of neurotoxicity, asthenia, thrombocytopenia, lymphocytopenia, and other side effects of CT [33,89,119,122] and may enhance the therapeutic effect of anticancer drugs [89,104,118,120,121,134]. If this is so, it would be possible to adjust needed doses of drugs or prescribe their continuous and longer cycles. In addition, the potential of Mel could be further explored for supportive and palliative therapy, at least to improve a patient’s quality of life [135].

In clinical studies by Lissoni et al., patients have taken oral Mel at high daily doses (≥20 mg) [70,71,88,89,118,119,120,121,125,136]. A favorable effect on the survival of patients with prostate cancer has been reported at a dose of 3 mg/day [137]. Thus, the question of optimal doses of Mel remains open. The oral route of administration may not be optimal because of lower bioavailability and individual variability [138,139]. Studies on relatively small groups of patients with terminal stages and different tumor localizations deserve attention and are provided in reviews [33,122]. In a study of 30 patients with metastatic CRC, the authors observed a higher percentage of disease control in the group of patients receiving irinotecan in combination with Mel [121]. The combined use of Mel with CT may increase the survival of patients with advanced non-small cell lung cancer (NSCLC) and gastrointestinal cancer [118,120,127,140]. Adjuvant Mel treatment has no effect on disease-free survival in patients with resected early-stage NSCLC but may confer prolonged survival in those with late-stage disease [141]. Notably, the positive effect of Mel on overall survival in prostate cancer patients has also been noted only in patients with an unfavorable prognosis [137]. In general, the benefit of treatment with Mel should be considered in individual cases, rather than in populations, with an assessment of the dynamics of tumor markers. For example, the oncostatic effect of Mel treatment may depend on the expression of the MT1 receptor subtype in the tumor, as has been shown in a case report involving hormone-refractory prostate cancer. Mel can affect the PSA level, which has been assessed as temporary stabilization of the disease [142].

The effect of Mel on immunocompetent cells and cytokine production in cancer patients remains poorly studied. An earlier preliminary study found differences in circulating cytokine levels, including decreased IL-6 levels, in patients with advanced solid tumors after 30 days of Mel administration (10 mg/day) [143]. Mel supplementation (20 mg/day) after 6 months did not affect NK cell cytotoxicity or phenotype, nor did it affect blood levels of inflammatory cytokines in patients with NSCLC [141]. The combination of IL-2 and Mel may stabilize the disease and increase the survival of patients with metastatic tumors [125,126,127,144]. The survival of patients with solid tumors and brain metastases who received RT of the brain was studied. Mel intake had no positive effect on survival compared with historical controls with a median of 4.1 months. The authors noted that the efficacy of Mel in advanced cancer is needed in further studies before a conclusion can be reached on the utility of Mel in this setting [145]. The effect of Mel on survival has not been found in patients with advanced NSCLC receiving CT. The authors note that studies are needed using a longer period of observation and a large sample size since their study included patients with a potentially poor prognosis with a median survival of 7.3 months [146].

The use of Mel supplements to improve sleep has increased significantly over the past decade in both healthy populations and cancer patients, which may be of interest for observational studies. The ability of Mel to improve sleep and quality of life has attracted attention as insomnia, anxiety, depression, pain, and other symptoms often accompany cancer. According to a meta-analysis, Mel might decrease chronic pain [147]. Bedtime Mel intake significantly reduced fatigue and the risk of depressive symptoms, improved quality of life and sleep, and increased expression of clock genes in women with breast cancer [148,149,150,151,152]. However, in patients with NSCLC who took Mel at a daily dose of 20 mg, no beneficial effects were observed in improving quality of life or reducing symptoms or side effects of CT and RT [141]. The beneficial effects of Mel users on quality of life and symptoms have not been confirmed by a systematic review and meta-analysis as well [153]. The authors believe that the main sources of heterogeneity leading to inconsistent results may be related to different types of cancer as well as methods and duration of treatment; therefore, large-scale randomized controlled trials including combinations, dosage, and duration of Mel administration deserve further study [153].

It should be taken into account that population-based studies are highly heterogeneous, and this poses a challenge for verifying the utility of integrative treatment. Stratification of the patients could reduce heterogeneity and inter-individual variability. However, the complex network of multifactorial interactions and abnormalities in cancer is a challenge despite large-scale analyses and can lead to inconsistent results. Currently, there is heterogeneity in the accumulated bioinformatics, which is due not only to inappropriate stratification, bias, quality of trials, or methods of assessment but also to the influence of numerous factors leading to different results in response to treatment. High heterogeneity may be due to tumor pathology itself, i.e., type, tumor localization, stages, gradation, genome instability and mutation, molecular and metabolic characteristics, and the type of treatment, doses, duration, bioavailability, and individual response to treatment associated with genetic polymorphism [154]. Moreover, concomitant diseases, the use of other drugs, physiological and psychosocial factors, and the composition of the resident microbiota may also affect the outcome of both CT and integrative treatment. In a critical multilateral analysis of reviews on the topic, “Melatonin and health,” the authors concluded that more systematic research is needed to understand and establish the connection between Mel and specific aspects of health [155]. Considering the safety and beneficial multifunctional effects of Mel as well as the decrease in its production with age [114,117,156,157,158], it is advisable to grant priority to clinical studies of Mel for the integrative treatment of cancer, in CT-mediated organ dysfunction, and in comorbid inflammatory and chronic diseases. Clinical studies of Mel and antioxidants in breast cancer are presented in a review [159]. Another patient-centered methodology, namely, the N-of-1 study design, is discussed in Section 4.3.

#### 4.1.3. Safety and Bioavailability of Exogenous Melatonin

Mel intake is safe even in high doses, as mild to moderate adverse events have been recorded [104,160,161]. Studies with prespecified low risk of bias criteria for meta-analysis did not find an increase in serious adverse events or associated treatment discontinuation. Oral administration of Mel causes minor and short-term adverse events that can likely be avoided or controlled by reducing doses and time. The most frequently reported adverse events are related to fatigue, mood, or psychomotor and neurocognitive performance. A few studies have noted adverse events related to endocrine and cardiovascular function. The authors believe that future long-term trials and studies of its interactions with endogenous hormones and pharmaceuticals will confirm its safety [162,163].

The bioavailability of Mel varies and depends on its absorption, metabolism, and elimination. In oral administration, the bioavailability varies from 9 to 33% and is approximately 15%. A higher bioavailability of Mel has been recorded in its penetration through the mucous membranes of the nose and mouth [104,138,139]. The pharmacokinetics of rectal and other routes of administration of Mel have also been described [164]. Physiological concentrations of Mel in blood serum, measured at night in the elderly, are 0.10–0.51 nM. The use of Mel in pharmacological doses (0.4 and 4 mg) causes a multiple increase in its average maximal serum concentration to 1.75 and 17.24 nM, respectively, but Mel is rapidly eliminated, with a half-life of 45 min [138,165]. It should be noted that some herbal preparations can significantly inhibit the metabolism of Mel, increasing concentration and time of elimination [166]. Mel supplements may contain quantities other than those listed on the product label or may contain serotonin because, unlike medications, supplement production is not strictly controlled. In some countries, Mel is available only with a prescription and is considered a drug.

### 4.2. Integrative Approach to Cancer Treatment

Research in the field of cancer therapy is mainly focused on the eradication of cancer cells. Combined oncotherapy aimed at vulnerable tumor targets can be more efficacious compared to monotherapy [167]. The complete eradication of cancer cells is a challenge today because of their molecular heterogeneity, mutations, plasticity, and self-renewal and because of the resistance of cancer stem cells to CT. Metronomic CT, which is referred to as low-dose continuous CT administration with minimal drug interruption, can affect the phenotype of nonproliferating dormant cells through antiangiogenic, immunomodulatory, and other mechanisms [12,168]. Studies in this field need to provide evidence on the safety and efficacy of comparative trials and identifying suitable patients [169,170]. Some medications are worth attention as off-label drugs for repurposing to oncology since they may exert an influence on cancer-associated signaling and metabolic pathways through epigenetic mechanisms causing tumor dormancy (a long period of latency), differentiation, or communicative reprogramming [9,10,11,171,172]. Disseminated tumor cells can persist in a dormant state for years, or even decades, after treatment but remain a source of cancer recurrence. New strategies are being considered to prevent relapse by maintaining or eradicating dormant tumor cells [173]. Reprogramming of tumor systems (anakoinosis) using epigenetically active drugs to convert malignant tumor cells into a less aggressive state is considered a future biotherapeutic strategy for managing tumor resistance and inducing long-term remission [11,172]. The interaction of Mel with various types of stem cells is of increasing interest in terms of reprogramming cancer stem cells. The results in non-transformed stem cells cannot be generalized to cancer stem cells, as the observed effects of Mel are often opposite in non-tumor and tumor cells [174]. Mel regulates abnormal aerobic glycolysis, gluconeogenesis, the pentose phosphate pathway, and lipid metabolism inherent in the metabolic program of cancer cells as well as transcription factors (HIF-1α and p53) [175]. Thus, biological therapies create new opportunities to slow down further cancer progression by changing the pro-tumoral imbalance in the microenvironment of residual cancer cells after primary tumor resection. Biotherapy may be useful for patients who refuse or discontinue CT and RT due to adverse effects as well as for mitigating adverse effects.

#### 4.2.1. Zinc and Selenium

The outcome of cancer patients and the effectiveness of therapy depend on the patient’s nutritional status [54]. Zinc and Se regulate numerous pathways in cancer and immune cells [176,177]. The mechanisms of the anticancer activity of zinc and its binding proteins are carried out through the stimulation of immune cells, the production of cytokines, the regulation of zinc-dependent enzymes, transporters and metallothioneins, apoptosis, transcription factors, gene expression, and signaling and through the influence on differentiation, proliferation, migration, inflammation, bioenergetic, metabolic, and other ways [176,178,179,180]. On the other hand, cancer cells need zinc to resist apoptosis through the mechanism of zinc-mediated caspase inhibition [176], and some zinc finger proteins (transcription factors) can both inhibit and promote cancer progression [180].

The bioavailability of zinc compounds depends on many factors, including gastrointestinal disturbances, inflammation, age, and type of diet (presence of absorption inhibitors in food) [181]. Zinc intake may improve liver function and reduce the risk of hepatocellular carcinoma, and its higher intake reduces the risk of colorectal and esophageal cancer [181,182]. The risk of mortality is significantly higher in laryngeal cancer patients with the lowest serum zinc levels compared with the highest [183]. Zinc deficiency has been found to be an independent predictor of gynecological cancer recurrence [184]. Experimental clinical studies of small groups showed a beneficial effect of zinc supplementation on the overall survival of patients with advanced nasopharyngeal carcinoma [185]. A critical review of pooled studies of zinc supplementation during cancer treatment noted a beneficial effect on mucositis after RT but not on CT-induced side effects. Overall, data from studies of larger cohorts rather argue against an effect of zinc on overall or disease-free survival, but the authors note heterogeneity in reported results [186]. Increased zinc intake during CT of patients with CRC may increase superoxide dismutase activity without affecting markers of oxidative stress [187]. An optimal intake of zinc could restore the normal immune response and reduce the risk of infection in immunocompromised cancer patients. However, the optimal immunostimulatory dose of zinc has not been determined. Moreover, an excess amount of zinc supplementation may cause immunosuppressive effects [176,178].

Se is an essential micronutrient, and its deficiency is harmful to human health [188]. The anticancer potential of Se is realized through various mechanisms, including redox balance and protection against ROS-mediated DNA damage, influence on gene expression, apoptosis, and cell cycle arrest, promotion of DNA repair, prevention of invasion and metastasis of tumor cells, and stimulation of the immune response [38,188]. Se compounds and their metabolites can act as antioxidants or pro-oxidants. Dietary organic Se, which is more bioavailable than its inorganic compounds, is incorporated into proteins to form selenoproteins, the latter functioning as oxidoreductases, synthetases, and Se transporters [38,189,190,191].

The anticancer activity of Se compounds and the synergistic effect with anticancer drugs observed in preclinical studies have prompted further clinical trials, alone or with standard therapy, to develop appropriate types, doses, and schedules [39,190,192,193,194,195,196,197,198]. Intake of Se (200 μg) in the form of Se yeast for 6 months may improve metabolic profiles and increase the percentage of regressed tumors, as has been shown in a small number of patients with cervical intraepithelial neoplasia [196].

Epidemiological studies have shown an increased incidence of cancer in populations with low Se intake and low plasma Se levels [193]. It has been reported that serum Se levels inversely relate to the risk of colorectal adenomas, severity of inflammatory bowel disease, and CRC [199]. The low level of Se in the serum of patients with colorectal and breast cancer is associated with advanced disease and a lower survival rate [40,200,201]. However, the chemopreventive effect of Se status on cancer risk and Se intake in clinical trials is still ambiguous and may be due to the heterogeneity of studies [38]. Cancer patients are often deficient in Se, and its supplementation is considered a promising option for radioprotection in RT without compromising the effectiveness of RT. No toxicity has been reported at doses of 0.3–0.5 mg/day, but it is recommended that Se status be determined in patients prior to RT [202,203]. High doses of sodium selenite can influence tumor chemoresistance and reduce the side effects of CT and RT, and tolerable doses of up to 5 mg have been studied in patients with advanced cancer [193]. In a Phase I study, doses up to 33 mg of sodium selenite as a single dose prior to RT were noted to be tolerable [204]. Some protocols have noted the safety and tolerability of high doses of sodium selenite, but the manifestation of its reversible toxicity still requires the selection of an appropriate dose and duration of treatment [193,194,197,202]. Naturally occurring organic Se compounds appear to have fewer side effects and fewer systemic effects compared to those reported for inorganic Se [177]. Methylselenocysteine and selenomethionine have been studied in clinical trials mainly in terms of their chemopreventive role in cancer [205]. Information on some Se compounds undergoing Phase I–III clinical trials is presented in review [190].

Se is a vital micronutrient for immune cells, and its deficiency impairs innate and adaptive immune responses. The benefits of Se supplementation for boosting immunity against pathogens, vaccination, or cancer in the general population have not yet received definitive support and need further comprehensive study, including redox mechanisms and the regulatory role of selenoproteins [191,206]. Due to a small safety window, excessive Se intake is still a subject of debate [188]. Increased intake of Se along with vitamins and minerals may be associated with an increased risk of mortality among patients with prostate cancer [207]. Thus, Se supplementation is beneficial for Se-deficient patients but requires proper monitoring of its levels since supranutritional doses might have adverse effects [208].

#### 4.2.2. Vitamin D3

Assessment of vitamin D status is based on the determination of its metabolite 25-hydroxyvitamin D (calcitriol, 25(OH)D). Calcitriol, an active hormonal form of vitamin D, has been extensively studied in preclinical models and is well represented in a review [209]. Briefly, its anticancer activity is mediated by various signaling pathways, including the regulation of numerous genes via the vitamin D receptor, inhibition of proliferation, angiogenesis and metastases, induction of apoptosis, autophagy and differentiation, anti-inflammatory and antioxidant effects, and effects on cancer-associated stromal cells, cancer stem cells, and immune cells in the tumor microenvironment [209,210].

A chemopreventive effect of vitamin D intake and an inverse relationship between circulating 25(OH)D levels and the risk of colorectal adenoma and CRC have been reported in a large systematic review and meta-analysis [211]. CRC patients are often deficient in circulating 25(OH)D, and higher levels of 25(OH)D are associated with better survival [19,211,212,213,214]. Some authors do not support a chemopreventive effect of vitamin D supplementation in patients with colorectal neoplasms [215]. Despite encouraging experimental and epidemiological observations, discrepancies have been shown in the therapeutic effect of vitamin D supplementation and its influence on mortality in CRC patients [209,212]. Most reported observations are heterogeneous due to poor stratification of patients by cancer pathology. For example, preliminary results from the AMATERASU randomized trial suggest that vitamin D supplementation may improve survival in a subgroup of patients with p53-positive tumors of the digestive tract or poorly differentiated adenocarcinoma [216,217]. In addition, the effects of vitamin D may depend on the dose and individual differences such as bioavailability, vitamin D-related metabolism, vitamin D receptor genotype, and body mass index [209,210,218,219], which are usually overlooked and may be the cause of inconclusive results. Thus, the multifaceted effects of vitamin D and its dysregulated metabolism and function in cancer represent a challenge for population-based studies and require well-designed clinical trial schedules based on patient stratification, response to treatment at appropriate doses, monitoring of the vitamin D metabolites, and determination of the vitamin D receptor genotype.

#### 4.2.3. Polyphenols

Plant-derived polyphenols are widely distributed and present in human food. The anticancer potential of polyphenols is well documented in preclinical models and has been examined for integrative treatment. Molecular mechanisms include modulation of signaling pathways, antioxidant and prooxidant activity, induction of apoptosis, autophagy, and cell cycle arrest; inhibition of proliferation of cancer cells, angiogenesis, invasiveness, and metastasis; and modulation of the immune response and anti-inflammatory effects [1,56,220,221,222,223]. The use of plant-derived polyphenols with anticancer drugs may reduce their toxicity and adverse effects or reverse the drug resistance of cancer cells [20,224]. However, caution should be exercised when using herbal products and CT drugs simultaneously. There is a risk of their interaction through the mechanisms of inhibition or induction of enzymes (the cytochrome P450 superfamily that metabolizes endogenous substrates and drugs) as well as through the effect on transport proteins (ATP-binding cassette drug transporters), which leads to a change in the pharmacokinetics of drugs [55,56,225]. Polyphenols could reduce intestinal and systemic inflammation through beneficial modification of the microbial community, which some authors consider a missing link in the mechanism of action of poorly bioavailable dietary polyphenols [56,226]. However, this concept requires detailed study since the action of polyphenols is not limited to pathogens [56].

The use of green tea extract after polypectomy may be beneficial for the prevention of colorectal adenoma [227,228]. Green tea consumption has been reported to reduce the risk of colorectal and other cancers, but evidence for its effect on cancer prevention and mortality has not been confirmed or has been limited to the highest category of green tea consumption compared to the lowest [45]. Despite promising results from preclinical studies, clinical studies have been inconclusive, which can be explained using high doses of polyphenols in preclinical models that appear to be beyond the reach of humans through diet and supplements [226]. In addition, clinical studies and meta-analysis of the association between plant polyphenol intake and the risk of developing CRC have revealed inconsistent results, which may be explained by low bioavailability, individual differences in the metabolism of polyphenols, their metabolic transformation depending on the composition of intestinal microbiota, and other factors [56,223,224,226,229,230]. For example, a nutrikinetic study of the oral administration of green tea polyphenols demonstrated interindividual variation in plasma concentrations, which could explain the partially contradictory effects observed in different populations or diseases [231].

Epigallocatechin gallate (EGCG) is one of the main green tea polyphenols, the anticancer activity of which has been confirmed in preclinical models [232]. Some clinical trials have shown that EGCG is generally well tolerated and may mitigate the harmful effects of RT [233]. Like other polyphenols, EGCG has poor bioavailability [232], but its use at high doses may evoke hepatotoxicity in rare cases. There are recommendations for users of green tea products to control liver function by ALT and AST [234]. The combination of EGCG with a high dose of Mel may alleviate or reduce the risk of possible EGCG hepatotoxicity [235]. In the present clinical case, the liver function of the patient was monitored by the markers ALT and AST, the values of which remained within the upper limits. Regarding an integrative approach to treatment, it is advisable to study such combinations that target vulnerable areas inherent in cancer and act through different mechanisms. Mel combined with some phytochemicals may have a complementary effect on tumor angiogenesis [13]. The enhanced oncostatic effect of Mel in combination with EGCG on cancer cells can be explained by the fact that the action of both occurs through different mechanisms [236].

Taxifolin (dihydroquercetin) is a polyphenol found in conifers (Siberian and Dahurian larch, French maritime pine, and others), in tamarind and milk thistle seeds, and in plant products (fruits, vegetables, oils, wines, etc.). The anticancer activity and mechanisms of taxifolin continue to be studied. Its dose-dependent ability to inhibit the viability, proliferation, migration, and invasion of cancer cells as well as to induce apoptosis through various mechanisms, including the Wnt/β-catenin signaling pathway, has been noted [237,238,239,240,241,242]. Taxifolin can prevent the development of obesity-induced hepatic steatosis, fibrogenesis, and tumorigenesis in a mouse model [243]. The protective efficacy of taxifolin against cisplatin-induced oxidative organ damage has been demonstrated in rodents [244,245,246]. In addition, taxifolin can inhibit P-glycoprotein-mediated transport activity in cancer cells, thereby reversing their multiresistance to chemotherapeutic agents. In this regard, taxifolin shows greater efficacy than green tea polyphenols and may increase the cytotoxicity of chemotherapeutic agents [247]. Taxifolin has a safety profile but low bioavailability [248]. Like other polyphenols, its concentrations and doses used in preclinical models have been significantly higher than those that could be achieved in clinical use.

#### 4.2.4. Aspirin

The beneficial effect of aspirin in cancer can be explained by various mechanisms, including a reduction in metastatic spread and the incidence of venous thromboembolism (anti-inflammatory and antiplatelet-mediated action). These mechanisms are associated with the inhibition of cyclooxygenases COX-1 and COX-2, which are necessary for the synthesis of prostanoids [63,249,250,251]. Low doses of aspirin (75 mg/day) can prevent PLTs from binding to tumor cells, thereby inhibiting cancer cell proliferation and metastasis via PLT-derived signals [10]. COX-independent mechanisms, such as induction of apoptosis and blocking of the WNT-TCF pathway, are less studied [5,10], and discussion of this topic is beyond the scope of this review. According to observational studies, regular use of aspirin can reduce the risk of recurrent colorectal adenomas and cancer mortality, but the benefit of aspirin for cancer is still controversial [252,253,254,255]. The use of aspirin may increase the risk of gastrointestinal and cerebral bleeding; however, discontinuation of aspirin intake may increase the risk of vascular diseases [250]. Co-administration of aspirin with Mel may be beneficial in protecting the gastric mucosa from aspirin-induced damage [256].

### 4.3. N-of-1 Study Design

The methodology of the N-of-1 trial represents a holistic approach to health accommodated to a patient-centered model rather than a drug-centered model [257,258]. The N-of-1 study design allows a treatment to be tailored to the patient depending on his response. Engaged patients can provide feedback and be more compliant towards the following recommendations. Recently, the N-of-1 trial design was upgraded to a high level of evidence for an individual’s treatment efficacy, placing this study design on par with a meta-analysis [259]. In terms of complementary treatment, N-of-1 studies are more suitable for treating symptoms of chronic and stable diseases [259]. N-of-1 trial designs can optimize treatment for patients, including those who cannot be included in RCTs due to uncertainty about the appropriate treatment pathway or for other reasons beyond the study criteria [259,260]. In N-of-1 studies, each participant serves as his or her own control. The duration of the N-of-1 study depends on the withdrawal period, i.e., washout to avoid carryover effects from the previous intervention and to properly assess the effect of the next intervention [260].

The power of N-of-1 studies depends on the number of clinical parameters measured rather than the number of participants and can help speed up cancer drug development, given its smaller number of participants and lower cost. One of the major limitations of N-of-1 studies is the inability to generalize the observed effects to the patient population. The effects of N-of-1 trials can be pooled and assessed in a stratified population, but this potential is considered a future prospect. Currently, N-of-1 studies remain heterogeneous, are useful as preliminary clinical data, and require a modified and standardized approach to estimate the efficacy of drugs used in oncology [257]. Dynamic studies and N-of-1 designs on individual patients have limitations for evidence-based medicine due to the influence of possible concomitant diseases requiring additional intervention. Repeated interventions using placebo and withdrawal periods demonstrating replication of the effect may reflect a causal relationship. Comparison and aggregation of serial studies of homogeneous groups with the same design may provide a more objective assessment and support for the hypothesized effect of integrative treatment.

**Figure 5:**
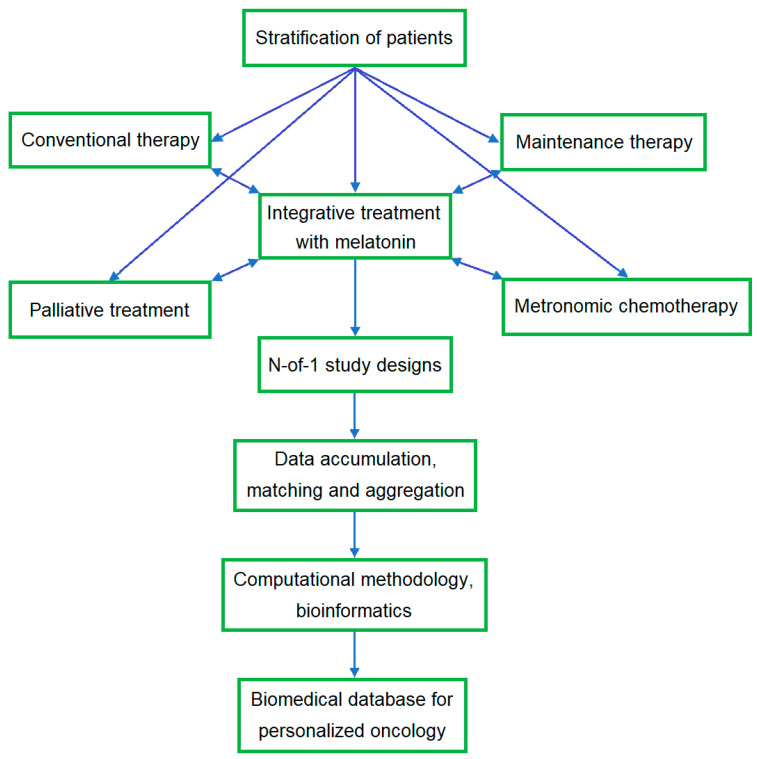
Conceptual scheme of integrative treatment with Mel in patients with gastrointestinal cancer for clinically relevant N-of-1 studies, which include monitoring stratified patients using imaging, blood tests, and questionnaires. Imaging studies: computed tomography, magnetic resonance imaging, positron emission tomography, ultrasonography, and angiography. Blood tests: monitoring of hematological and biochemical parameters, including markers of inflammation and tumors, markers of organ function and hemostasis, control of Mel production, and deficiency of essential micro/macroelements and vitamins. Stratification of patients: stratification by molecular pathology to select eligible participants and reduce heterogeneity; molecular–genetic and histological profiling of primary tumors and metastatic specimens and liquid biopsies for analysis of circulating tumor DNA and tumor cells; and the use of oncogenomics and next-generation sequencing technology. Conventional therapy: surgery, CT, RT, immunotherapy, and targeted therapy; stratification of patients by the response to therapy when comparing effectiveness vs. adverse effects based on pharmacogenomics [262]. Integrative treatment with Mel: the influence of Mel on effectiveness, adverse effects, quality of life, relapse, and survival when used in combination with conventional, maintenance, metronomic, and palliative therapies. Maintenance therapy is repeated treatment to prolong remission. Metronomic CT: a new type of CT in which anticancer drugs are administered repeatedly in lower doses over a long period to treat cancer with fewer adverse effects; metronomic CT is noteworthy as an N-of-1 study, designed either as a stand-alone treatment modality or in combination with Mel. Palliative treatment in combination with Mel to improve quality of life by normalizing sleep, correcting circadian rhythms and organ function, and relieving symptoms of chronic diseases and complications associated with cancer.

Adequate personalized therapy involves studying the molecular genetic profile of the patient’s tumor and response to therapy. Evaluation of the Mel potential in integrative treatment using N-of-1 studies may involve a variety of designs, including its influence on CT effectiveness and adverse effects, tumor-associated systemic effects, and quality of life as well as tumor recurrence and survival (Figure 5). The N-of-1 study protocols may be considered for an RCT, similar to the trial evaluating the effectiveness of Mel in insomnia in children with neurological disorders [261]. Like Mel, natural supplements may also be examined using N-of-1 trials.

## 5. Conclusions

Numerous preclinical studies of adjunctive treatment of cancer using natural products have now been published, but these remain outside the scope of clinical practice. In clinical studies, special attention is drawn to non-toxic natural products that mitigate the harmful effects of CT and RT but do not affect the effectiveness of conventional therapy. Mel is considered a biomodulator that influences various pathways inherent in the initiation and progression of cancer. Preclinical and preliminary clinical studies suggest a dual effect of Mel in cancer treatment, as it may increase the effectiveness of anticancer drugs and mitigate their harmful effects on the body.

The present case report analyzes the dynamics and the well-being of a patient taking Mel for a long time, focusing on abnormal clinical parameters and adverse effects of CT. The use of Mel improved well-being and sleep, and its side effects were well tolerated. The patient’s condition during CT combined with Mel was satisfactory, and the polyneuropathy was temporary and manifested as a reaction to cold. The residual manifestation of polyneuropathy was a decrease in the sensitivity of the toes and soles. No effect of Mel supplements on thrombocytopenia was found, which may be associated with the patient’s predisposition to a lower PLT count. Inflammatory biomarkers (NLR, LMR, and PLR) varied after CT combined with Mel but remained within accepted favorable limits. The number of lymphocytes remained within the reference values and increased after surgery and during CT in combination with Mel. Postoperative levels of CEA and CA 19-9 were within the reference range. The PSA level decreased during CT in combination with Mel but increased after CT and especially to the end of Mel withdrawal and decreased after resuming Mel intake. The level of D-dimer increased during CT with Mel and then gradually decreased, while the APTT remained near the upper limits and then increased. A significant increase in the PLT count was observed as a reaction to treatment with azithromycin, including the Mel withdrawal period.

Regarding some recommendations and the results of preclinical and clinical studies, the patient took food supplements as well as aspirin after CT. Patients may use various combinations of antioxidants and nutritional supplements based on generally accepted information and their subjective perception and well-being. There are potential risks of simultaneous use of different medicines and herbal products, mainly related to their interactions, so the choice of treatment option should be made under the supervision of healthcare professionals and nutritionists [263].

Verification of the utility of Mel and natural supplements for integrative treatment in clinical settings is challenging due to the high heterogeneity and inter-individual variability of population-based studies. An alternative approach to a parallel group of RCTs may be N-of-1 study designs, with the potential to minimize costs and resources [264]. Multivariate and aggregated N-of-1 study designs are suitable for advancing biomedical and translational studies in precision medicine and can also provide participants with real-time care [260]. N-of-1 studies deserve attention to optimize the integrative treatment of complications caused by cancer and anticancer therapy and could be included in future medical research programs focused on collaboration with patient partners [265]. Retrospective and prospective studies of cancer patients treated with Mel in dynamic and N-of-1 designs based on protocols and questionnaires will help advance integrative treatment using Mel.

## Figures and Tables

**Table 1:** Dynamic study of clinical parameters.

Clinical Parameters	Before Surgery	After Surgery	During and after CT	54 Months after Diagnosis	Reference Ranges
Neutrophils *	3.90	3.08	2.00–1.69–1.85	3.46	2.20–7.60
Lymphocytes *	1.38	1.50	1.85–2.61–2.05	1.96	1.00–3.60
Monocytes *	0.71	0.61	0.70–1.01–0.61	0.64	0.20–1.00
Platelets *	164	145	75–227–96–110	142	150–400
NLR	2.83	2.05	0.77–0.91–1.61–0.92	1.77	<2.72
LMR	1.94	2.46	2.58–3.36–2.43–4.79	3.06	>2.83
PLR	118.8	96.7	40.5–106.1–41.0–96.3	72.5	<219.0
C-reactive protein, mg/L	<1.0	11.0	<1.0	1.0	<5.0
Eosinophils *	0.05	0.30–0.18	0.22–0.34–0.20	0.16	0.10–0.40
RBC, number × 10^12^/L	4.35	3.83	3.72–3.39–3.56	4.08	4.50–6.00
Hb, g/L	136	116	113–117	128	130–180
CEA, μg/L	6	1.4	1.2–1.7	1.70	<3.8 **
CA 19-9, kU/L	13	6	7–9	8	<27
PSA, μg/L	5.70		4.62–4.17–7.61–5.28	6.40	<4.00
D-dimer, mg/L		0.61	1.86–1.43–0.43		<0.50
APTT ***, s	38.7	42.9	36.6–39.2–44.2		28.6–38.2

* Number × 10^9^/L. ** Value for non-smokers (Synlab). *** Activated partial thromboplastin time. Other clinical parameters were within the reference ranges or showed minor deviations from limits.

## Data Availability

The data are not publicly available for privacy reasons.
